# The Cause and Prevention of Sudden Death after Fifty1Read at the thirtieth annual meeting of the Medical Association of Central New York, held at Buffalo, October 19, 1897.

**Published:** 1898-09

**Authors:** Sydney A. Dunham

**Affiliations:** Buffalo, N. Y. 180 West Chippewa Street


### THE CAUSE AND PREVENTION OF SUDDEN DEATH AFTER FIFTY.[1]

### THE HEART, KIDNEYS, LUNGS AND BRAIN, ALCOHOLISM AND SUICIDE BRIEFLY CONSIDERED.

By SYDNEY A. DUNHAM, M. D., Buffalo, N. Y.

IT IS the purpose of the writer to consider, under the above title, death which has occurred after a brief illness, from one to two days in adults, who did not consider themselves sick, who were busy

1. Read at the thirtieth annual meeting of the Medical Association of Central New York, held at Buffalo, October 19, 1897.

in their professional and commercial life, but in whom, nevertheless, a close medical examination would have disclosed in one or more of the vital organs of the body pathologic changes which have gradually developed from natural causes or predisposition, or have been acquired by disease, accidents or excesses.

Bichat gives this difference between death from old age and death from sudden seizure—namely, that "in the former death commences at the circumference and ends at the center, while in the latter, death begins at the centers of vitality and gradually extends to utmost bounds."

Death may be expected, but sudden death is a serious thing and when it could have been prevented is allied to suicide. "How old are you?" is an every-day question. The physician answers most correctly in saying that "a man is as old as his arteries."

Balfour, in an interesting volume on the senile heart, says that "age must be measured by tissue changes and not by years, because those tissue changes which mark the progress of development from the cradle to the grave, intensify after middle life all the dangers of acute diseases and by accentuating any latent organic weakness or structural defects, inherent or acquired, often cause those to die from age who have scarcely began to think themselves old."

An adult dies suddenly, the coroner is called and the first thought and word is "heart disease," according to the ancient and out of date phrase *cor ultimum moriens*. As to the order in which the vital organs have been found to be diseased, in cases of sudden death, the heart stands first, and the coroner's snap diagnosis is correct in a majority of cases. Sudden death is more frequent after fifty than before, and increases with age in all cases except those of suicide. The four principal organs in the order of their frequency by which they cause sudden death and those which I shall consider in this paper are: the heart, kidneys, brain and lungs.

*Cause of sudden death from diseased heart*.—Among the immediate causes revealed at an autopsy may be found rupture of the heart walls—principally the left ventricle, the contributing causes being fatty or fibroid degeneration, circumscribed myocarditis, necrosis following thrombosis or embolism of the coronary arteries; rupture of an aneurism of the arch of the aorta, perforating into either auricle or thoracic cavity, rupture of coronary arteries, due to atheroma, filling the pericardial cavity with blood; adherent pericardium, due to old attacks of pericarditis; a chronic valvular disease, due to endocarditis and certain congenital malformations of the heart, which have gone on to middle life before producing sudden death.

*Cause of sudden death from diseased kidneys.*—It is only in recent years that our knowledge of kidney disease is sufficient to consider them among the organs that cause sudden death. After heart lesions the kidneys are the next structure that are likely to show disease at the post mortem.

Sir George Johnson is credited with the statement which is today the expression of the experience and sentiment of the leading physicians that a cirrhotic kidney is a common occurrence in those who eat and drink to excess.

Apoplexy, convulsions, coma and edema of the larynx may be the immediate cause of death, but upon examination of the kidneys the chief cause or the principal lesion is revealed. In chronic Bright's disease a cirrhotic or contracted kidney is the form most commonly recognised as producing sudden death. Brouardel, of Paris, author of Death and Sudden Death, believes that sudden death in cases of gout always has a kidney origin. In gouty patients, when the function of the kidneys suddenly becomes altered, associated with degenerative changes in the circulatory system, we may expect impending danger. The first premonition the patient may have of this painless, deceptive disease may come like a thief in the night or the blow of the highwayman in the form of sudden death. Business and professional men have seen their associates falling out of the ranks as if by an enemy in ambush. The clergyman says it is a dispensation of Providence ; the physician, at heart, believes that it is a dispensation of ignorance or avarice, and knows that a careful medical examination would have disclosed the disease, and by treatment could have averted the impending catastrophe. Chronic diffuse nephritis is the most insidious disease that can occur after middle life. It is fatal and gives short notice when not discovered early.

Apoplexy, pneumonia and Bright's disease are the common causes of death after fifty years of age. The statistics for Buffalo show how close they run in order of frequency. In 1894, apoplexy, 91 ; Bright's disease, 44 ; pneumonia, 46. In 1895, apoplexy, 89 ; Bright's disease, 59 ; pneumonia, 59. In 1896, apoplexy, 98 ; Bright's disease, 76 ; pneumonia, 72. In 1897, apoplexy, 103 ; pneumonia, 107 ; Bright's disease, 102.

In seeking life insurance many an applicant has been told of some unknown malady which they must dispose of before being accepted.

The physician today would paraphrase a verse of Blair's as follows:

"How shocking must thy summons be, O Death."
(To him who did not think himself sick.)
"Who counting on long years of pleasure here."
(Is quite untreated for that world to come.)

*Cause of sudden death from brain disease.*—The brain is held responsible for many things that go wrong with many other vital organs, but still it is the most helpless in defending itself after the others have suffered decay. The brain is so situated, and its blood supply so regulated, that it was prearranged to be the last organ in the body to suffer decay or lose its functions. The brain itself seldom originates disease, but is continually being bombarded by foreign substances from the other organs of the body in the form of emboli, which shut off the nutrition and the uremias and toxemias paralysing its functions. While brain tumors and abscesses are not infrequent, thrombosis and embolism of the cerebral blood-vessels are the more common causes, with chronic endocarditis and arterio-sclerosis contributing. Apoplexy and intracranial hemorrhage may show immediate cause, with aneurism and endarteritis, alcoholism and syphilis contributing. Here is an illustration of a medico-legal case of common occurrence in large cities: In a prize fight or social combat, or in making an arrest, one man receives a blow upon the head or face from which death ensues; the other is held upon a charge of murder. The autopsy shows a chronic pachymeningitis with the dura-mater adherent, endarteritis and miliary aneurism of the cerebral arteries, together with the marks of syphilis or the history of chronic alcoholism.

While post mortem examiner of Erie County in 1890 a case of this kind came before the coroner for decision. The autopsy revealed, as described above, the diseased condition of the cerebral vessels with a diffuse hemorrhage into the corpora striatum, optic thalami and ventricles. The cause of death was given as accidental and from natural causes. It was considered that under such physical conditions sudden death could have taken place at any time by any great mental emotion or physical effort. Any circumstance which increases the intracranial pressure under such conditions would be likely to cause death. Certainly a case of this kind should not go to trial on circumstantial evidence alone.

*Cause of death from organs of respiration.*—Pulmonary apoplexy, edema of the larynx, pneumonia, pneumothorax, hydrothorax and hematothorax, are some of the fatal lesions.

A physician of this city while under treatment for rheumatism at the Warsaw sanitarium, three years ago, died suddenly of edema of the larynx. He had been troubled with rheumatism for some time. A woman under my care died of pneumothorax, which was the result of septicemia.

Infarction of the lung with a following pneumonia commonly cccurs in acute or chronic diseased conditions of the endocardium. Hydrothorax may be one of the fatal complications of kidney lesions with inflammation of the pleura and other serious surfaces. Pathologic conditions of the respiratory organs, causing sudden death, are largely dependent upon heart and renal disease, *e. g.*, pneumonia and pulmonary congestion due to diseased heart and pulmonary edema, due to diseased kidneys.

### PREMONITORY SYMPTOMS AND NOTES OF WARNING.

Men after fifty should ponder over Herbert Spencer's definition of life, as it will guide them in adopting preventive measures which are reliable. He speaks of life as "the continuous adjustment of internal relations with external relations." When men and women after fifty years of age have been informed (findeth wisdom) that some previous illness, *e. g.*, typhoid fever or other infectious disease, pneumonia or rheumatism has altered the function and structure of the heart or kidneys, they should be moderate in eating and drinking, avoid violent effort—both physical and mental—secure the advice of the family physician and then "length of days will be in their right hand, and in their left, riches and honor."

While low degenerative changes in the vital tissues of the body may not be cured, they can easily be alleviated and frequently arrested.

*Premonitory symptoms referable to the heart.*—Precordial anxiety or uneasiness, bradycardia, tachycardia arrhythmia and angina pectoris are among the earlier symptoms and when associated with organic changes, such as fibrous and fatty degeneration of the myocardium, coronary sclerosis, dilatation and hypertrophy, are premonitions of danger, and the prognosis is based on the response which these conditions give to proper treatment. Angina pectoris is more commonly found in males, because arterio-sclerosis is more frequent and the first attack may prove fatal. Balfour states that "the essential lesion of the senile heart is a weakened myocardium and dilatation is the first stage of cardiac change."

The beginning of the fatal heart lesion was probably an endocarditis or endarteritis occurring a score or more of years previous and gradually following came sclerosis of the valves, rendering them incompetent, and then the insidious involvement of the coronary arteries, shutting off the nourishment to the heart muscle.

The more remote causes still will be found in articular rheumatism, pleurisy, pneumonia, pericarditis, malaria, alcoholism and syphilis. The general malaise, anorexia, anemia and toxemia of today, showing deficient elimination, without treatment, will blow the flame to irreparable damage in later years.

Many people are like an old ship, they are all right in fair weather, but go down when they encounter a storm. The only safety for the captain of such a vessel is to consult the weather bureau or the doctor.

*Premonitory symptoms referable to the kidneys.*—The signs of distress and danger will be noticed in the slow, continued headache, occasional dizziness, dimness of vision (neuro-retinitis) sleeplessness, difficulty in breathing, muscular twitching and gastric disturbances. A closer examination reveals the urine diminished in quantity, of low specific gravity, usually a trace of albumin, casts may or may not be present, dilatation and hypertrophy of the heart.

While hypertrophy of the left ventricle of the heart and cirrhotic kidney are co-incident, Gull and Sutton deny any direct casual connection between the two, but hold that the two conditions are the result of one general affection of the arterial system, which they call arterio-capillary-fibrosis; that this condition primarily affects the arterioles and invades the other organs—heart, kidneys, lungs, brain, spinal cord and so forth—as a wide spread cachexia, which has its base in the vascular system.

As in the earlier stages of chronic Bright's disease, so in the senile degeneration of the heart and arteries, there may be no pathognomonic symptom until the action of the heart is altered or the secretion of the urine changed in quantity and quality.

We cannot be dogmatic in asserting when such a termination will occur, but as physicians we should be able to give a uniform, positive prognosis in such terms that the patient will heed them. It will be more conducive to happiness and longevity if it were the custom to have a physical examination with a chemical and microscopical one of the urine made semi-annually in adults after they have reached the age of fifty. We have learned in the last few years that all contagious and infectious diseases are preventable, and it will be but a short time before the constitutional diseases of the aged will be considered preventable to a degree greater than at present. Contagious and infectious diseases of early life are preventable because they are understood and the manifestations are external and receive proper treatment, while the constitutional diseases of adults are not as well understood because their manifestations are internal, without alarming the patient until they are advanced.

Age, gout, alcoholism, syphilis and infectious diseases, according to all the authorities reviewed upon the subject, are the chief among the causes which produce the structural changes in the vital organs already named. These chronic, deceptive and largely preventable diseases, due to such poisons as uric acid, alcohol and syphilis which predispose the race chiefly to sudden death are not properly treated.

Alcoholism, having been so long considered a vice, many physicians are slow to look upon it as a disease. In chronic cases where punishment has failed, treatment from one to six months has proved a success. And more than this it has proved a diseased condition in the alcoholic. When punishment is necessary, as with beginners, it should be severe. Our present law for punishing drunkards is too lenient to have the desired effect. Drunkenness is too great an offence against decency, law and order, to go without a severe penalty.

They should be treated from one to three months as other nervous diseases are treated. Alcoholism produces more sudden deaths including accidents, than any other disease, and would when treated greatly reduce the death-rate from suicide.

Syphilis when treated with the thoroughness and exactness which the treacherous disease requires, would prevent aneurism, which so frequently is the cause of sudden death; also suicide, which is dependent upon cerebral gumma or endarteritis. As soon as the external manifestations have disappeared the patient quits the doctor, notwithstanding the admonitions to the contrary.

When the moral force is lacking, the state in other things lends a hand. Something should be done to compel young men to continue treatment for syphilis until dismissed by the physician, or have his name kept in a book provided for that purpose by the board of health; such names to constitute a list of ineligibles for matrimony. A contract between patient with syphilis and the physician for treatment until cured for from $100 to $300 would help toward keeping up treatment for two or three years, which is required.

*Causes of sudden death from alcoholism.*—Alcoholism is more frequently the cause of sudden death than is reported, because some do not want the stigma in the family recorded, and because they die of alcoholism some are refused burial in consecrated ground. It may cause death by producing a congestion of all the principal organs of the body, with no appreciable lesion in any.

A middle-aged gentleman of this city, of fine physique, carrying over $100,000 life insurance, died suddenly one morning while dressing. Autopsy showed considerable congestion of the brain, lungs, liver, kidneys, stomach and slight atheroma of the aorta. A chemical examination of the contents of the stomach revealed no poison and the death certificate was filed, giving as the cause of death no appreciable lesion sufficient to produce death. It was afterward learned that he was a very hard drinker, but not becoming intoxicated easily it was not known to many of his friends. The evening preceding his death he used alcoholic beverages freely. Nervous inhibition is the only way to account for some deaths from alcoholism.

This paper would hardly be complete without some attention to suicide which, according to the better authorities, is on the increase.

Lawrence Irwell, of this city, who has given time to looking up statistics, says that "suicide increases from childhood to the age of fifty-five, and then declines, and that it is due to racial deterioration." In the Christian civilisation, where religion never looks upon it as an honorable thing to do, and where the act is punishable by law, suicide ought not to be on the increase. I would prefer to consider suicide the result of racial dissipation, rather than of racial deterioration Alcoholism is the preponderating cause of suicide, according to French and American authorities.

If the incipient stages of melancholia and other mental troubles were as well understood as the earlier stages of consumption and other infectious diseases, suicide would be more generally prevented. Why certain mental diseases lead to suicide has never been ascertained only as far as suicidal mania does not differ from other manias, such as kleptomania and dipsomania, the uncontrollable impulse is never removed by punishment and only prevented by watchful attendance and treatment.

*Post mortem always essential after sudden death.*--In 1890, an able bodied man, over fifty, died suddenly while in his chair at dinner. The coroner presented to me, for signature, a certificate of death which gave heart disease as the cause. Preferring to make an autopsy, the heart was examined with the other viscera, and found normal. Before examining the brain I decided to open the trachea and just beneath the larynx could be seen fibers of meat pressing between the vocal cords, and upon opening the larynx a large piece of steak was found beneath the epiglottis, well wedged into the glottis. The cause of death was changed from heart disease to apnea or asphyxia.

A young man who had died apparently in good health while watching a funeral procession, and whose friends afterward thought it was a bad omen, revealed at the autopsy the left thoracic cavity filled with blood, due to rupture of a thoracic aneurism. The young man had had syphilis one and a half years, but without treatment. There were no external sores showing the history of syphilis. An autopsy is not only necessary to ascertain the cause of sudden death, but to counteract the influence of superstition, charlatanism and christian science. A middle-aged man, under the care of the christian scientists, was taken suddenly worse and a physician was called who administered to the dying man in the absence of his wife. When the wife returned she accused the doctor of killing her husband, therefore the physician would give no certificate of the cause of death without an autopsy. An almost complete mitral stenosis showed cause of death.

As believers and workers in preventive medicine we should recommend that adults after fifty seek medical advice regularly, at least annually, as a matter of business conducive to longevity; that the life insurance companies establish the precedent of requiring a medical examination annually of all policy-holders over fifty years of age and whose policy exceeds $2,000; that the treatment of alcoholism and syphilis should be made compulsatory by law; that physicians try and agree in their examinations and be uniform in their prognosis of individuals in their declining years, and thereby to hasten the day when people at maturity will have the ever increasing confidence in the medical profession which their progressive science based upon higher medical education deserves.

180 West Chippewa Street.

---

To clean rusty instruments immerse them over night in a saturated solution of stannous chloride. Rub dry with chamois after rinsing in running water and they will be of a bright silvery whiteness.—*Medical Age.*

